# Cytotoxic Activity of Polyphenol Extracts from Three Oregano Species: *Hedeoma patens*, *Lippia graveolens* and *Lippia palmeri,* and Antiproliferative Potential of *Lippia graveolens* against Two Types of Breast Cancer Cell Lines (MDA-MB-231 and MCF-7)

**DOI:** 10.3390/molecules27165240

**Published:** 2022-08-17

**Authors:** Marilyn S. Criollo-Mendoza, Rosalío Ramos-Payán, Laura A. Contreras-Angulo, Erick P. Gutiérrez-Grijalva, Josefina León-Félix, Claudia Villicaña, Miguel A. Angulo-Escalante, J. Basilio Heredia

**Affiliations:** 1 Centro de Investigación en Alimentación y Desarrollo, AC. Carretera a Eldorado Km 5.5, Col. Campo el Diez, Culiacán CP 80110, SI, Mexico; 2 Facultad de Ciencias Químico Biológicas, Universidad Autónoma de Sinaloa, Calzada de Las Américas Norte 2771, Col. Burócrata, Culiacán CP 80030, SI, Mexico; 3 Cátedras CONACYT, Centro de Investigación en Alimentación y Desarrollo, AC. Carretera a Eldorado Km 5.5, Col. Campo el Diez, Culiacán CP 80110, SI, Mexico

**Keywords:** oregano (*H. patens*; *L. graveolens*; *L. palmeri*), polyphenols, antioxidant activity, antiproliferative, breast cancer

## Abstract

Oregano infusions have traditionally been used to treat some diseases related to inflammation and cancer; also, some species have shown antiproliferative activity on cancer cell lines, for example, colon and liver, and this has been attributed to its phytochemical profile, mainly its phenolic compounds. This study aimed to evaluate the cytotoxicity and antiproliferative potential of the polyphenols-rich extracts (PRE) of the oregano species *H. patens*, *L. graveolens,* and *L. palmeri* on breast cancer cell lines. The PRE of the three oregano species were obtained from dried leaves. The extract was characterized by determining antioxidant activity, total phenols content, and identifying the profile of phenolic acids and flavonoids by chromatography UPLC-MS/MS. Furthermore, the cytotoxicity of the extracts was evaluated in vitro on a non-cancer cell line of fibroblast NIH3T3 and the antiproliferative potential on the breast cancer cell lines MDA-MB-231 and MCF-7. *L. graveolens* showed the highest antioxidant capacity and significantly inhibited the proliferation of MCF-7 and MDA-MB-231 cells at non-cytotoxic concentrations in normal cells, with a similar effect to that cisplatin in MDA-MB-231 cells. Therefore, the polyphenol-rich extract from *L. graveolens* showed the greatest potential to guide future research on the antiproliferative mechanism of action.

## 1. Introduction

Oregano is a common name from a group of species with characteristic smell and flavor; at least 61 species of 17 genera belonging to six different botanical families are known by this name [1,2]. In México, approximately 40 species of herbaceous plants are recognized with this name; *Hedeoma patens* M.E. Jones, *Lippia graveolens* Kunth, and *Lippia palmeri* S. Watson are among the most commercialized [3]. Traditionally, they have been used as a condiment in food preparation by adding dried leaves, while infusions have been used to treat some diseases related to inflammation, such as cough, headache, and toothache, among others [4,5,6]. Oregano has been reported to have antioxidant potential and anti-inflammatory activity; these properties are attributed to their phytochemical profile, mainly to its phenolic compounds [7,8,9,10,11]. Oxidative stress and inflammation are related to the onset and development of comorbidities of many chronic diseases such as diabetes, cardiovascular diseases, and cancer. In this sense, the appearance of some cancers has been linked to chronic inflammation, and this knowledge has allowed for new anti-inflammatory therapeutic approaches for the prevention and treatment of cancer [12]. In this sense, different oregano species have been associated with antiproliferative activity against various types of cancer cells [9,13,14,15,16]; additionally, some compounds such as flavonoid and phenolic acid derivatives of oregano species have been related to the ability to inhibit the proliferation of some cancer cells of the colon, liver, and breast [13,15,17,18].

Breast cancer is one of the most important and studied worldwide because it affects 1 in 10 women [19]. It also represents one of the highest mortality rates compared to other types of cancer and is the second most diagnosed, mainly in developed countries (around 55% of the global burden). However, the incidence rates are increasing in developing countries [20]. Conventional treatments, due to their low cell specificity, have harmful side effects on patients since, in addition to damaging cancer cells, they affect normal cells, decrease the patient’s quality of life, and can increase the risk of death [21]; so currently, many investigations are focused on finding alternative treatments as adjuvants or as chemopreventive agents focused mainly on plant derivatives [22,23]. For this reason, it is important to investigate the bioactive potential of the Mexican oregano species *H. pattens*, *L. graveolens* and *L. palmeri*, since there are no previous reports in the literature of the cytotoxic activity of these species in normal cells, as well as evidence of antiproliferative activity of the species *L. graveolens* on breast cancer. Therefore, this study aimed to evaluate the cytotoxic activity of polyphenol-rich extracts (PRE) from three species of oregano as well as the potential to inhibit cell proliferation in breast cancer lines (MDA-MB-231 and MCF-7).

## 2. Results and Discussion

### 2.1. Antioxidant Activity of PRE of Three Oregano Species 

The antioxidant activity of the three oregano species is shown in Table 1. *L. graveolens* showed the highest inhibition of the 2,2-diphenyl-1-picrylhydrazyl (DPPH) radical, followed by *L. palmeri* and *H. patens*. Similar behavior was observed in the oxygen radical absorbance capacity (ORAC) assay results, finding that the species with the highest antioxidant activity was *L. graveolens*. Statistical analysis showed a significant difference between the three species evaluated (*p* < 0.05). It has been reported that the antioxidant capacity of oregano samples is partly due to its high content and distribution of phenolic compounds [24,25]. The results obtained in this work are greater than those of Gutierrez-Grijalva et al. [26] for the same oregano species. In the literature, the change in the content and the phytochemical profile of these and other species of plants have been related to certain factors, such as the manipulation of nutrients and the stress to which the plant is subjected, among others. In addition, the biotic and abiotic factors stimulate the synthesis of elicitors, which could result in a greater production of secondary defense metabolites [27,28].

### 2.2. Total Phenolic Content of PRE of Three Oregano Species

The Folin-Ciocalteu assay was carried out to measure the total phenolic content in the oregano species; however, it is often considered a method to evaluate a sample’s total reducing capacity. The highest content of total phenols was found on *L. graveolens* spices with 143.87 mg of GAE/G, followed by *L. palmeri*, and finally, *H. patens*, which presented the lowest content with 99.58 mg of GAE/g (Table 1). The statistical analysis showed a significant difference between the three species evaluated. According to the results, these oregano species show a higher total phenols content than those reported for these same species (40.74, 51.26, and 22.87 mg of GAE/ g for *H. patens*, *L. graveolens*, and *L. palmeri*, respectively) [26]. Despite being the same oregano species, this can be attributed to the fact that they were obtained from different harvested samples. The conditions to which the plant was exposed, its growth, collection, and storage can affect both the antioxidant amount and phytochemical profile [7]. Similar results were found when comparing our results with other oregano species such as *Origanum vulgare* [29].

### 2.3. Phenolic Compounds Profile Identified in PRE of Three Oregano Species

For the analysis and identification of compounds, the molecular ion and the fragments obtained in the spectra of each oregano species were taken as reference and compared with the data of compounds reported for other oregano species and in the Mass bank database of North America (MoNA). Our results coincide with that shown for other oregano species such as *Origanum dictamus*, *O. vulgare,* and *Lippia micromera*, highlighting the presence of structures belonging to the subgroups of phenolic acids, flavones, flavonols, and flavanones [30,31,32].

Among the phenolic compounds identified in the three oregano species are hydroxycinnamic and hydroxybenzoic acid derivatives such as gallic, vanillic, and caffeic acid, and flavonoids such as eriodictyol, luteolin-7-glucoside, baicalein, and luteolin (Table 2). In the specific case of *H. patens*, mainly flavone-type compounds were identified. For instance, apigenin-7-neohesperidoside was identified only in this oregano species; chlorogenic acid was also detected. Regarding the *L. graveolens* species, quercetin-3-O-hexoside was the only compound identified in this oregano species compared to the other two analyzed. However, this oregano species had previously identified compounds such as eriodictyol, naringenin, phloridzin, and luteolin [8,33]. On the other hand, *L. palmeri* showed a profile of compounds very similar to the other two analyzed species; a similar profile was observed compared to *Lippia origanoides*, *Majorana hortensis,* and *Origanum acutidens* [14,34,35].

### 2.4. Cytotoxicity of PRE of Three Oregano Species on Non-Cancer Fibroblast Cells and Antiproliferative Activity of the Extracts on Breast Cancer Cells

**Figure 1:**
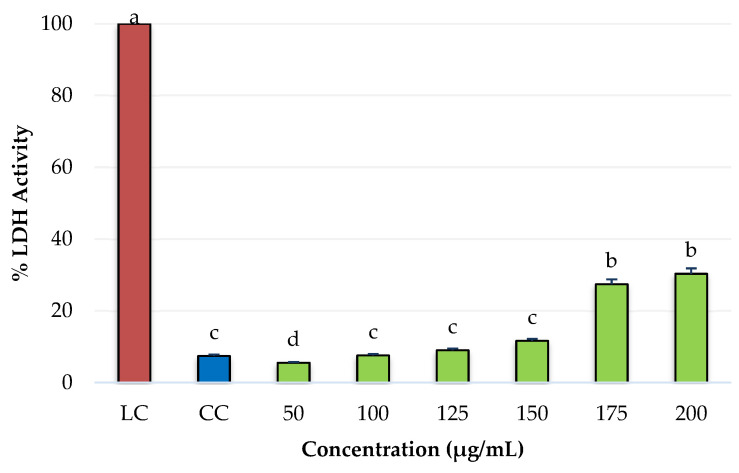
Cytotoxicity of PRE from *H. pattens* (HP), *L. graveolens* (LG), and *L. palmeri* (LP) on normal fibroblast cells (NIH3T3), expressed as a percentage of lactate dehydrogenase activity (LDH). LC (Lysis Control), CC (Cell Control). Values with a different letter are statistically different (*p* ≤ 0.05). Results are expressed as means (n = 3) ± standard deviation (bars).

The results obtained from cytotoxicity of PRE of the three species of oregano are presented as a percentage of lactate dehydrogenase (LDH) enzyme activity as an indicator of cell death (Figure 1).

On the left side of Figure 1, a lysis control is shown with a 100% activity of LDH enzyme and a cellular control (cells without PRE). The maximum value activity was 25.24%, obtained at the 200 μg/mL concentration for the species *L. graveolens*, which did not significantly differ when the concentration was increased to 300 μg/mL. Regarding the cell control, concentrations of 300 μg/mL of *H. patens* and 200 and 300 μg/mL of *L. graveolens* showed a significant difference; these latter concentrations have a cell viability of 75% and can therefore be considered cytotoxic for normal NIH3T3 fibroblasts [36].

In this sense, the phenolic compounds present in the three oregano species contain in their structure some hydroxyl groups, as well as carbonyl groups. According to the literature, there is a positive correlation between the number and type of functional groups and the cytotoxicity because both carbonyl and free hydroxyl groups, mainly in the form of ortho-diphenol radicals, increase the cytotoxicity of compounds such as caffeic acid, rosmarinic acid, and luteolin-7-O-glucuronide that are present in our samples [14]. Furthermore, it has also been shown that ortho-diphenolic residues can contribute to the increase in toxicity by chelation and reduction in transition metals (Fe, Cu, Zn), causing the generation of harmful hydroxyl radicals [37]. This information could be related to the cytotoxicity of the oregano species due to the differences in its phytochemical profile.

**Figure 2:**
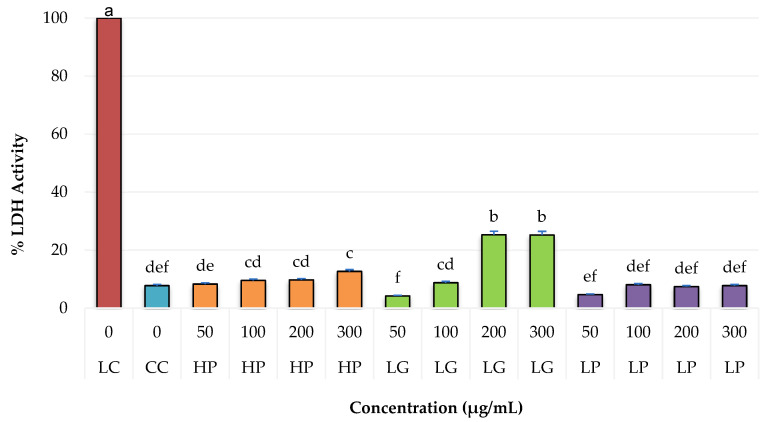
Cytotoxicity of PRE from *L. graveolens* (LG) on normal fibroblast cells (NIH3T3), expressed as a percentage of lactate dehydrogenase activity (LDH). LC (Lysis Control), CC (Cell Control). Values with a different letter are statistically different (*p* ≤ 0.05). Results are expressed as means (n = 3) ± standard deviation (bars).

For the antiproliferative activity of the PRE of oregano on breast cancer cells MDA-MB-231 and MCF-7, only *L. graveolens* extracts were tested because they showed greater potential when evaluating cytotoxicity. For this, we again analyzed the extract on NIH3T3 fibroblast cells to determine the maximum non-toxic concentration in non-cancer cells, showing antiproliferative activity in breast cancer cells. Concentrations of 125, 150, 175, and 200 μg/mL of PRE were tested, finding a decrease in viability of 25% from 175 μg/mL (Figure 2), for which it was decided to evaluate a concentration lower than 175 μg/mL on breast cancer cells to evaluate its antiproliferative potential.

**Figure 3:**
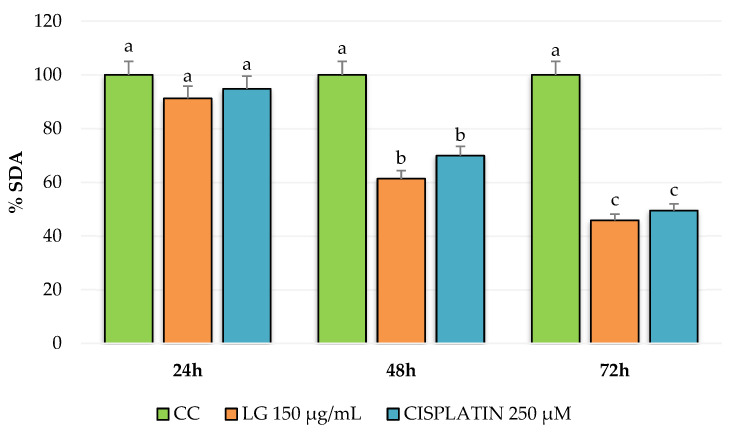
Antiproliferative potential of PRE of *L. graveolens* on MDA-MB-231 breast cancer cells expressed as a percentage of succinate dehydrogenase activity (%SDA). Values with different letters are statistically different (*p* ≤ 0.05). The results are expressed as means (n = 3) ± standard deviation (bars).

**Figure 4:**
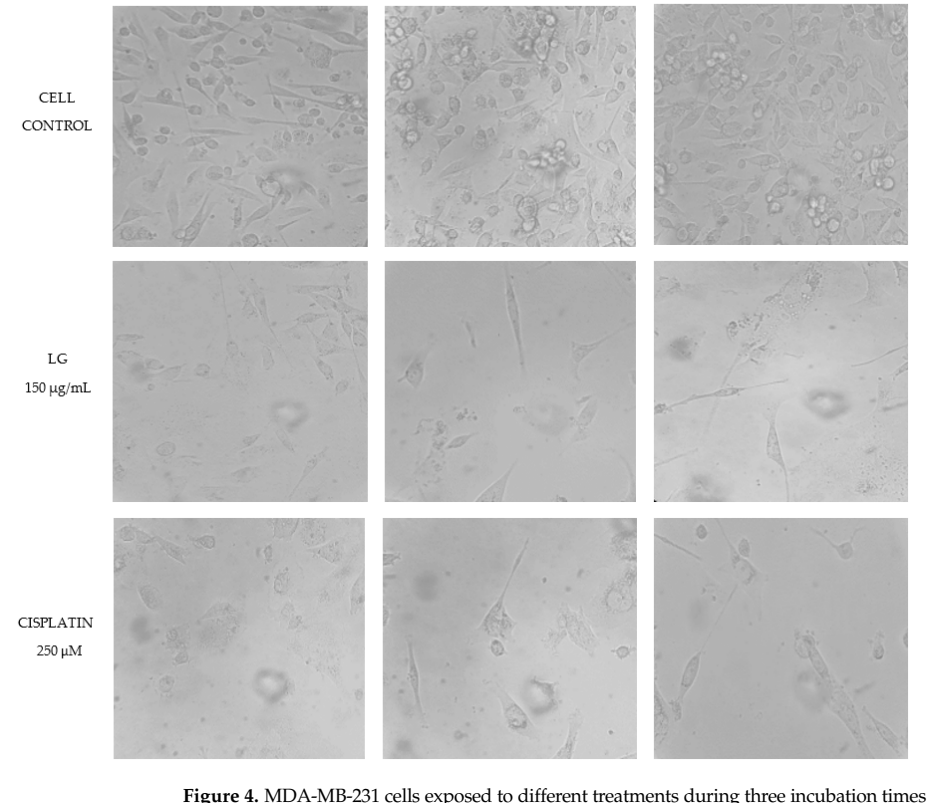
MDA-MB-231 cells exposed to different treatments during three incubation times (24, 48, and 72 h). Cell control represents cells without treatment. Images obtained under the microscope (40×).

The results obtained when evaluating the PRE of *L. graveolens* in the MDA-MB-231 breast cancer cells are shown in Figure 3. A decrease in cell viability is observed compared to the control group (cells with culture medium only) in the three evaluated incubation times: 24, 48, and 72 h. However, in the results obtained at 24 h, the LG extract at 150 µg/mL and cisplatin at 250 µM did not significantly differ from the cellular control. On the contrary, in the evaluations at 48 and 72 h, there was a decrease in cell viability of approximately 60% and 45%, respectively. Additionally, the effect of LG on the reduction in cell viability was dependent on the exposure time. According to the statistical analysis, no significant difference was observed between the antiproliferative activity shown by the extract and the reference drug used in this experiment, which we were able to confirm by watching the cells under a microscope at each incubation time. According to Figure 4, after 24 h of treatment with both PRE of *L. graveolens* and cisplatin, a decrease in the number of cells present in the medium can be observed, compared to the cell control, a behavior that is conserved as time progresses of incubation.

**Figure 5:**
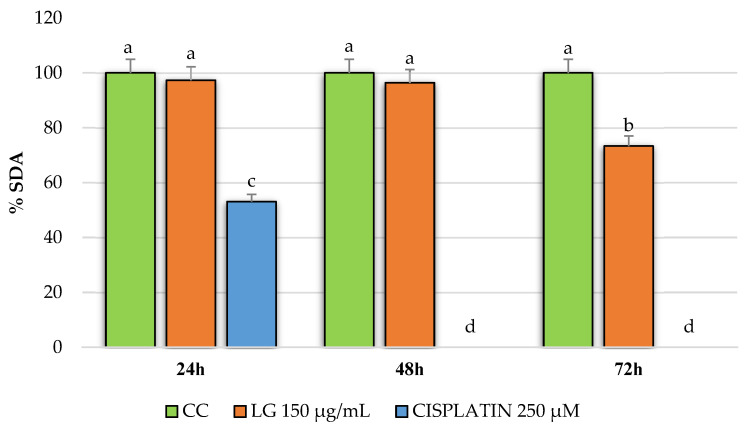
Antiproliferative potential of PRE of *L. graveolens* on MCF-7 breast cancer cells expressed as a percentage of succinate dehydrogenase activity (%SDA). Values with different letters are statistically different (*p* ≤ 0.05). The results are expressed as means (n = 3) ± standard deviation (bars).

**Figure 6:**
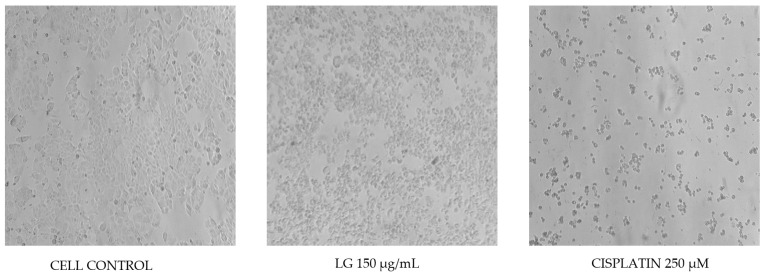
MCF-7 cells exposed to different treatments during 72 h incubation. Cell control represents cells without treatment. Images obtained under the microscope (40×).

On the other hand, Figure 5 shows the results obtained from MCF-7 breast cancer cells treated with PRE of *L. graveolens* at 150 µg/mL and cisplatin at 250 µM for 24, 48, and 72 h. We found no significant difference between the extract and the control group at 24 and 48 h since the decrease in cell proliferation was minimal, contrary to the effect of cisplatin, which showed a reduction of nearly 50% at 24 h. In comparison, at 48 h, it completely inhibited the proliferation of this type of cell. In the 72 h treatments, the PRE of *L. graveolens* reduced cell proliferation to approximately 73%. At the same time, cisplatin completely inhibited cell proliferation, which can be seen in the images from the microscope in Figure 6. We realized that besides the decrease in cells count, as compared to the control, the PRE of *L. graveolens* caused damage to the normal morphological characteristics of this type of cells, since the presence of cellular contraction was noted.

In some in vitro studies, compounds such as quercetin and baicalein, found in PRE of *L. graveolens*, have shown the ability to inhibit the growth of breast cancer cells without causing negative effects on normal cells [38,39,40], which coincides with the results obtained in this work.

The activity showed by PRE of *L. graveolens* is similar to that reported for flavonoid-rich pecan nut extract on MCF-7 cells [41]. In addition, a decrease in cell proliferation was also observed when evaluating some flavonoids isolated from propolis, such as apigenin and luteolin on MDA-MB-231 cells (effect dependent on the dose and treatment time) [42]. This may be related to their structural characteristics, such as the arrangement and number of hydroxyl groups that contain, the double bond in C2-C3, the presence of the ortho-catechol group, and the hydroxyl group in C3 [43,44]. Furthermore, the antioxidant compounds present in the PRE of *L. graveolens* (phenolic acids and flavonoids) can act as cellular antioxidants by protecting DNA from damage caused by oxidative stress, as well as by inhibiting lipid peroxidation and reducing associated inflammation to an excess of free radicals. They can also chelate metals, thereby preventing the catalytic reactions of some free radicals, thus preventing or delaying inflammation and cancer development [45,46,47,48].

An important aspect to mention about the results obtained in this work is the potential selectivity of PRE of *L. graveolens* on the MDA-MB-231 cells because we were able to observe that the treatment at 24 h with 150 µg/mL of the extract resulted in a decrease in cell proliferation more remarkable compared to that observed in MCF-7 cells, where the inhibitory effect of the extract could be observed up to 72 h of treatment (Figure 4 and Figure 6). Regarding the comparison of the extract with the reference drug used, we could also observe significant differences in both cell lines; MCF-7 cells were more sensitive to 250 µM cisplatin treatment compared to MDA-MB-231 cells. However, the effect of cisplatin on these cells was very similar to that shown by PRE of *L. graveolens*, an important result because the concentration of PRE evaluated in this experiment did not present cytotoxicity in non-cancer cells of NIH3T3 fibroblasts. Therefore, this represents an opportunity to contribute to developing new drugs from natural sources. However, it is important to continue investigating the mechanism of action by which this activity is being carried out.

In this sense, it has been reported in the literature that some compounds, such as quercetin and rosmarinic acid, have a high capacity to inhibit the activity of the aromatase enzyme by binding to estrogen receptors, which means that there is less estrogen available to stimulate the multiplication of hormone receptor-positive breast cancer cells, as is the case of the MCF-7 cell line [47,49,50].

On the other hand, the potential of flavonoids in treating different breast cancer types has also been related to their interaction with signaling pathways that contribute to the development of self-renewal and differentiation in the mammary gland, such as Wnt, Notch, and Hedgehog [51]. Flavonoids can also induce apoptosis by activating the caspase signaling cascade and activating effector caspases such as caspase-3 and caspase-7 and can increase the proapoptotic ratio of the Bax/Bcl-2 family of enzymes. It has also been reported that flavonoids can cause cell cycle arrest at various stages of the cell cycle, for example, G2/M, simply by acting on the activity of cyclins [52,53]. Thus, we suggest further studies must be performed using the PRE of *L. graveolens* to evaluate their potential antiproliferative mechanism of action associated with the content of these types of compounds.

## 3. Materials and Methods

### 3.1. Plant Material

*H. patens* M.E. Jones, *L. graveolens* Kunth, and *L. palmeri* S. Watson were collected in Surutato, Sinaloa (coordinates: N 25°51′6.2′′, W 107°34′56.6′′), Santa Gertrudis, Durango (coordinates: N 23°32′43.8′′, W 104°22′20.8′′) and Todos Santos, Baja California Sur (coordinates: N 23°27′26.1′′, W 110°14′0.77′′), respectively. Species identification was conducted at the Herbarium from the School of Agriculture at the Universidad Autónoma de Sinaloa. The identification catalog numbers for each species were *L. graveolens* FA-UAS-017005, *L. palmeri* FA-UAS-007551, and *H. patens* FA-003840.

### 3.2. Preparation of the Oregano Polyphenols Rich-Extracts (PRE)

The PRE from *H. patens, L. graveolens,* and *L. palmeri* were extracted according to Gutierrez-Grijalva, Angulo-Escalante, Leon-Felix, and Heredia [26], with slight modifications. First, a sample of 0.2 g oregano leaf powder was incubated with 10 mL of 80% methanol for 24 h without light. After incubation, the samples were centrifuged at 12,000× *g* for 15 min, and the supernatant was collected. Then, three replicates of each oregano were prepared and stored at −20 °C for the experiments.

### 3.3. Antioxidant Activity

#### 3.3.1. ORAC

For the ORAC assay, fluorescein as a fluorescent probe and AAPH (2,2′-Azobis 2-methylpropionamidine dihydrochloride) was used as a peroxyl radical generator. This assay was performed on a 96-well microplate with dark walls and a clear background [54]. First, an aliquot of 25 μL of PRE, 25 μL of a blank (75 mM phosphate buffer, pH 7.4), and 25 μL of a standard Trolox curve were added; then, the plate was placed in a Synergy HT microplate reader (Bio-Tek Instruments, Winooski, VT, USA) and pre-incubated at 37 °C for 15 min. The equipment dispensed in each well of the plate 200 μL of fluorescein 0.96 μM and 75 μL of 95.8 μM AAPH, initiating the reaction with this last. The fluorescence was measured every 70 s for 70 min with a 485 nm excitation filter and a 580 nm emission. The results were calculated using the linear regression equation of a standard Trolox curve of 6.25 to 125 (μmol TE/g) and the net area under the curve of the fluorescein loss. The values were expressed as μmol of Trolox equivalent per gram of sample (μmol TE/g).

#### 3.3.2. DPPH

The DPPH (2,2-diphenyl-1-picrylhydrazyl) scavenging capacity assay was carried out according to Karadag et al. [55]. First, in a 96-well clear microplate, 20 μL of 80% methanol solution (blank), 20 μL of PRE of each species, and 20 μL of a standard Trolox curve were added (0.05 to 1 mmol TE/g). Then, 280 μL of DPPH was added and left to incubate for 30 min without white light. After this time, absorbance was measured at 540 nm using a Synergy HT spectrophotometer Synergy HT, Bio-Tek Instruments, Winooski, VT, USA). The results were expressed as mmol of Trolox equivalent per gram of sample (mmol TE/g), and dilutions were prepared when needed.

### 3.4. Determination of Total Phenolic Content

The total phenolic content was determined using a modified Folin-Ciocalteu colorimetric method [56]. The procedure consisted of adding 15 μL of PRE in a 96-well microplate; then, 240 μL of distilled water and 15 μL of Folin-Ciocalteu reagent were added. The mixture was first incubated for 3 min, and 30 μL of 4N Na_2_CO_3_ was added; then, a second incubation was performed at 25 °C for 2 h. The absorbance was measured in a microplate reader spectrophotometer (Synergy HT, Bio-Tek Instruments, Winooski, VT, USA) at 725 nm using methanol 80% as blank. The calculations were performed using a gallic acid standard curve (from 0 to 0.4 mg/mL). The results were expressed as milligrams of gallic acid equivalents per gram of sample (mg GAE/g).

### 3.5. Identification of Phenolic Compounds by LC-ESI-QTOF-MS/MS

The identification of phenolic acids and flavonoids was performed in a UPLC Acquity class H (Waters) system, coupled to a mass analyzer G2-XS QToF Waters, quadruple, and time of flight (Waters Corporation, Santa Clara, CA, USA). The separation was performed with Acquity UPLC BEH C18 1.7 μm 2.1 × 100 mm column at 40 °C. The mobile phase consisted of phase A: acidified water with 0.1% formic acid and a phase B: acetonitrile, with a flow of 0.2 mL/min, with an injection volume of 2 μL. The gradient elution procedure was as follows: 0 min, 90% (A); 3 min, 70% (A); 9 min, 60% (A); 11 min, 50% (A); 12 min, 0% (A), 13 min, 0% (A); 15 min, 90% (A); and 17 min, 90% (A) [26]. The ionization of the compounds was performed by electrospray (ESI). The parameters were set as the capillary voltage of 1.5 kV, sampling cone 30, desolvation gas 800 (L/h), and temperature of 500 °C. Collision energies of 10, 20, and 30 V were used.

### 3.6. Cell Culture

All cell lines (NIH3T3, MDA-MB-231, and MCF-7) were acquired from the American Type Culture Collection (ATCC, Manassas, VA, USA). The cell lines were cultured as recommended by the suppliers in an incubator at 37°C with 5% CO_2_ until reaching the appropriate density for the tests. Culture media and fetal bovine serum were purchased from Gibco Life Technologies, (Thermo Fisher, Waltham, MA, USA). Penicillin-Streptomycin was purchased from Sigma-Aldrich, St. Louis, MO, USA.

### 3.7. Cytotoxicity Assay

The cytotoxicity of the PRE of oregano species was evaluated by the In Vitro Toxicology Assay Kit based on the activity of the lactate dehydrogenase enzyme (LDH) following the supplier’s recommendations (Sigma-Aldrich, St. Louis, MO, USA). In a 96-well sterile plate, 5 × 10^4^ NIH3T3 cells /well and concentrations of 50, 100, 200, and 300 μg/mL of PRE of each species were placed 24 h before the experiment and incubated at 37 °C with 5% CO_2_.

### 3.8. Antiproliferative Activity

According to the supplier’s recommendations, the antiproliferative activity was assessed by In-vitro Toxicology Assay Kit MTT (Sigma-Aldrich, St. Louis, MO, USA). Breast cancer cells line hormone-dependent MCF-7 and triple-negative MDA-MB-231 were plated in 96-well sterile plates at 2 × 10^4^ cells/well density. Concentrations of 150 μg/mL of PRE were added, and plates were incubated for 24, 48, and 72 h at 37 °C with 5% CO_2_. Cisplatin (250 μM) was used as the reference drug to compare the effect on the treated cells. The percentage of cell viability was expressed as a percentage of succinate dehydrogenase activity (SDA) and calculated as follows:

(1) $$\%\;\mathrm{SDA}=\frac{\mathrm{Absorbance}\;\mathrm{control}-\mathrm{Absorbance}\;\mathrm{sample}}{\mathrm{Absorbance}\;\mathrm{control}\;}\cdot 100$$

### 3.9. Statistical Analysis

All analyses were performed in triplicate (n = 3). Data were expressed as means ± SE. The statistical significance of differences among means was estimated by one-way analysis of variance (ANOVA) and Tukey test, using the statistical package Minitab 17 (Minitab Inc., State College, PA, USA). The statistical differences at the level *p* < 0.05 were significant.

## 4. Conclusions

The polyphenol-rich extracts of *H. patens* and *L. palmeri* did not show cytotoxicity on normal cells at the highest concentration. *L. graveolens* showed cytotoxic activity at 175 μg/mL and significantly inhibited in a time-dependent manner the proliferation of MDA-MB-231 and MCF-7 cells at a lower concentration (150 μg/mL). The PRE of *L. graveolens* showed the best potential in inhibiting the proliferation of MDA-MB-231 cells (compared to MCF-7 cells) and is equivalent to that shown by cisplatin, one of the drugs currently used for the treatment of breast cancer. The results obtained can be attributed to the presence of flavonols and some flavones, such as apigenin and luteolin, identified in this oregano species, which have already been reported in the literature with this bioactivity but through different mechanisms. Therefore, the PRE with the greatest potential to guide future research on the antiproliferative mechanism of action is obtained from the oregano species *Lippia graveolens*, which could represent an opportunity to contribute to the development of new drugs from natural sources. However, it is very important to continue investigating the mechanism of action by which this activity is being carried out. Moreover, the possible synergy between the identified compounds should be assessed in future research.

## Figures and Tables

**Table 1:** Results of total phenolic content (TPC) and antioxidant capacity (DPPH, ORAC) of PRE of three oregano species.

Oregano Species	TPC (mg of GAE/g)	DPPH (mMol TE/g)	ORAC (µMol TE/g)
*H. patens*	99.58 ± 0.42 ^c^	2140.89 ± 0.26 ^c^	575.42 ± 33.23 ^c^
*L. graveolens*	143.87 ± 1.29 ^a^	2523.67 ± 0.36 ^a^	3870.01 ± 27.41 ^a^
*L. palmeri*	114.30 ± 4.48 ^b^	2301.93 ± 0.21 ^b^	1365.54 ± 106.46 ^b^

Values with different letters are statistically different (*p* ≤ 0.05). The results are expressed as means ± standard deviation (n = 3).

**Table 2:** Phenolic compounds identified in PRE of three oregano species.

					Oregano Species		
MS	[M-H]-	Fragmentation Pattern	Tentative Identification	Classification	*H. pattens*	*L. graveolens*	*L. palmeri*
170.02	169.01	125.02 *, 168.83, 170.83	Gallic acid	Phenolic acids (hydroxybenzoic acid derivative)	+	+	+
354.09	353.09	179.03, **191.05**, 354.09	Chlorogenic acid	Phenolic acids (hydroxycinnamic acid derivative)	+		
168.04	167.03	108.02, 123.04, **152.01**	Vanillic acid	Phenolic acids (hydroxybenzoic acid derivative)	+	+	+
180.04	179.03	134.03, **135.04**, 178.84	Caffeic acid	Phenolic acids (hydroxycinnamic acid derivative)	+	+	+
288.06	287.05	**151.00**, 288.06	Eriodictyol	Flavanone	+	+	+
506.01	505.09	463.08, **506.09**	Quercetin-3-O-glucose-6′′-acetate	Flavonols		+	+
448.1	447.09	**448.09**, 449.10	Luteolin-7-glucoside	Flavone	+	+	+
462.08	461.07	**285.04**, 462.07	Kempferol-3-glucuronide	Flavonols	+		+
464.08	463.08	**300.02**, 302.02, 464.09	Quercetin-3-O-hexoside	Flavonols		+	
578.16	577.15	**269.04**, 270.05, 578.15	Apigenin-7-neohesperidoside	Flavone	+		
446.08	445.07	175.02, **269.04**, 446.08	Baicalin	Flavone	+	+	+
302.04	301.03	**302.03**, 303.04	Quercetin	Flavonols		+	+
436.13	435.13	**273.07**, 436.13	Phloridzin	Dihydrochalcone		+	+
286.04	285.04	**151.00**, 286.04	Luteolin	Flavone	+	+	+
270.05	269.04	151.00, **270.05**	Apigenin	Flavone	+	+	
272.06	271.06	**151.00**, 177.02, 269.04	Naringenin	Flavanone		+	+
360.08	359.08	**161.02**, 197.05, 360.23	Rosmarinic acid	Phenolic acids (hydroxycinnamic acid derivative)	+		+

* Numbers in bold correspond to the fragment of greater intensity.

## Data Availability

Not applicable.

## Sample Availability

Samples of *H. patens, L. graveolens and L. palmeri* are available from the authors.
